# Management of the Elderly Patients with High-Grade Serous Ovarian Cancer in the REAL-WORLD Setting

**DOI:** 10.3390/curroncol28020110

**Published:** 2021-03-07

**Authors:** Michalis Liontos, Alkistis Papatheodoridi, Angeliki Andrikopoulou, Nikolaos Thomakos, Dimitrios Haidopoulos, Alexandros Rodolakis, Flora Zagouri, Aristotelis Bamias, Meletios-Athanasios Dimopoulos

**Affiliations:** 1 Department of Clinical Therapeutics, Alexandra General Hospital, School of Medicine, National and Kapodistrian University of Athens, 11528 Athens, Greece; alkistispapath@gmail.com (A.P.); aggandrikop@med.uoa.gr (A.A.); fzagouri@med.uoa.gr (F.Z.); abamias@med.uoa.gr (A.B.); mdimop@med.uoa.gr (M.-A.D.); 2 Department of Obstetrics and Gynaecology, Alexandra General Hospital, School of Medicine, National and Kapodistrian University of Athens, 11528 Athens, Greece; nthomakos@med.uoa.gr (N.T.); dchaidop@med.uoa.gr (D.H.); arodolak@med.uoa.gr (A.R.)

**Keywords:** ovarian cancer, elderly, high-grade serous

## Abstract

Treatment of elderly patients with neoplasia is challenging. Age is a known prognostic factor in ovarian cancer but the optimal treatment of elderly patients has not been determined. We undertook a retrospective analysis to determine clinical practice in advanced-stage ovarian cancer patients older than 70 years of age. *Methods*: Medical records of women with high-grade serous ovarian cancer, stage III and IV were retrospectively analyzed. *Results:* A total of 735 patients were identified with a median age of 61.5 years. 22.4% among them were older than 70 years of age at diagnosis. First-line Progression-Free Survival (PFS) and Overall Survival (OS) were significantly worse in elderly patients in comparison to the younger ones [mPFS 11.3 months vs. 14.8 months, (*p* < 0.001) and mOS 30.2 months vs. 45.6 months (*p* < 0.001)]. However, elderly patients were characterized by worse ECOG-Performance Status and they were more frequently treated with Neoadjuvant Chemotherapy followed by Interval Debulking Surgery, while often they were more frequently denied debulking surgery compared to patients under 70 years of age. Moreover, elderly patients received more frequently monotherapy with platinum as frontline treatment. In contrast, there was no significant difference in the outcome of the debulking surgery in comparison to the younger patients or the frequency that gBRCA test was performed. Age over 70 years did not retain its significance for either Progression-Free Survival or Overall Survival when adjusted for all other reported prognostic factors. *Conclusions:* Elderly ovarian cancer patients have a worse prognosis. Comprehensive geriatric assessment should be performed for the optimal treatment of these patients.

## 1. Introduction

Ovarian cancer is the second most common gynecological cancer but the most lethal one, causing approximately 13,980 deaths worldwide in 2019 [1]. Its incidence increases with age, reaching its peak in the seventh decade of life [2]. In particular, in women older than 65 years of age, ovarian cancer incidence reaches 50.09 per 100,000, while in younger women its incidence is only 8.83 per 100,000 in the USA [3]. Due to population aging, it is estimated that ovarian cancer cases will increase in the following years.

Usually, women with ovarian cancer present with advanced diseases, such as FIGO stages III-IV [4]. The optimal initial management of these patients is primary debulking surgery followed by frontline systemic chemotherapy [5]. Patients unfit for surgery due to widely tumor spread will benefit from Neoadjuvant treatment with chemotherapy (NACT) and subsequently surgery, known as Interval Debulking Surgery (IDS) [5]. Moreover, all new patients should be offered testing for BRCA1/2 mutations, as it is estimated that the prevalence of these mutations is approximately 20% [6]. In case a mutation is identified, patients should be offered maintenance therapy with a polyadenosine diphosphate-ribose polymerase (PARP) inhibitor [7,8].

Treating elderly patients, however, seems quite challenging. These patients have often a complex medical history which limits their likelihood to undergo aggressive surgery [9,10]. Use of concomitant medication is also often associated with higher toxicity rates related to chemotherapy and schedule delays or reductions or even inability to receive standard chemotherapy [9,11,12,13]. Furthermore, patients over 75 years of age are inadequately represented in clinical trials [14,15]. Thus, it seems that there is an unmet medical need to define the optimal management of this population.

The aim of this retrospective study is to analyze the patterns of the everyday clinical practice of treating patients over 70 years of age with advanced-stage ovarian carcinoma.

## 2. Materials and Methods

### 2.1. Study Design and Patient Population

Patients with histologically confirmed high-grade serous ovarian cancer and advanced-stage disease (FIGO stage III or IV), treated in our institution from 1995 to 2018, were selected for analysis. All patients had given their written consent for the use of their medical data. The study was granted approval by our Institutional Review Board and was conducted accordingly to the Declaration of Helsinki.

All patients were divided into two groups based on their age; (a) elderly patients were defined as women over or at 70 years of age with advanced ovarian cancer, (b) younger counterparts were defined as women less than 70 years of age with advanced ovarian cancer.

Clinicopathological, treatment, and survival data were collected from patients’ records. More specifically, demographical data including patients’ date of birth, age at diagnosis, and date of first disease progression and/or death were collected. Type of surgery included primary or interval debulking and surgery outcome was defined as optimal or suboptimal. Tumor staging was performed in accordance with the FIGO staging system for ovarian cancer [16]. Data regarding chemotherapy regimens, namely, treatment with a combination of paclitaxel and carboplatin or carboplatin alone were also collected, as well as data concerning testing for BRCA mutations. Patients’ Performance Status was measured according to ECOG Scale Performance Status [17]. Overall Survival (OS) and Progression-Free Survival (PFS) were calculated as part of the survival analysis. These were calculated as the number of months from the date of cancer diagnosis since the date of death or confirmed disease progression, respectively [18].

### 2.2. Statistical Analysis

All data were coded and analyzed by a specifically designed database of the SPSS statistical package (SPSS Inc., Armonk, NY, USA) version 24. The Kolmogorov Smirnov test was used to assess the regularity of the data. The outcome of the debulking surgery was classified as optimal (absence of residual disease or residual disease below 1 cm) or suboptimal (residual disease more than 1 cm). Overall Survival (OS) was defined as the time between the time of diagnosis and the date of death from any cause. Progression-free Survival (PFS) was defined as the time between the time of diagnosis and the date of progression. Alive patients were censored at the date of the last contact. Kaplan–Meier estimates were used to describe and visualize the effect of categorical variables on Overall Survival and Progression-Free Survival [19,20]. Survival analysis was performed by Kaplan–Meier curves and survival differences between groups were estimated using the log-rank test. The estimation of the prognostic value of several variables with patients’ survival was made by Cox regression models. Multivariate Cox regression analysis was used to estimate the independent predictive value of the various factors in patients’ survival. All statistical correlations were considered significant in the case of *p* < 0.05.

## 3. Results

### 3.1. Baseline Characteristics

A total of 735 patients with advanced high-grade serous ovarian carcinomas were treated in our institution and were included in the analysis. Among them, 165 patients (22.4%) were 70 years of age or older at diagnosis and deemed as elderly for the current analysis. The median age in the whole population was 61.5 years (range from 24.7 to 89.2 years) and 74.2 years (70.0–89.2 years) in the elderly patients. Baseline characteristics of the entire population as well as of patients younger or older than 70 years of age are displayed in Table 1. There was a statistically significant difference between the two groups in all the examined clinicopathological characteristics with the exception of stage and frequency of testing for germline BRCA1/2 mutations. Especially, older patients had significantly worse performance status compared to their younger counterparts (*p <* 0.001) at baseline. Indeed, the majority of younger patients (82.6%) were fully functional at diagnosis, while one-third of the elderly (33.3%) were restricted from physically strenuous activities, as shown in Table 1.

### 3.2. Surgical and Medical Treatment

In everyday clinical practice, the management of advanced ovarian cancer differed significantly in older patients. More specifically, elderly patients were more frequently treated with Neoadjuvant Chemotherapy followed by Interval Debunking Surgery (IDS), while younger patients were mostly treated with Primary Debulking Surgery (PDS) (*p* < 0.001) (Table 1). In addition, a significant percentage of elderly patients (21/165, 12.7%) never received a debulking surgery, while this was seldom among younger patients (23/470, 4.0%). Younger patients did not receive cytoreductive surgery due to primary refractoriness to Neoadjuvant Chemotherapy (NACT), while in elderly patients apart from a progressive disease, other reasons were patient’s preference and poor performance status. The outcome of surgery was also worse in elderly patients. The percentage of suboptimal debulking increased in this subgroup of patients (Table 1).

Regarding medical treatment, optimal frontline chemotherapy consisting of platinum doublet was applied to almost all patients young than 70 years old. However, 15.7% of the older patients received carboplatin monotherapy, as were assessed as unfit for combinatorial treatment from treating physicians (*p* < 0.001). Furthermore, only 7.8% of the elderly patients received bevacizumab as part of their frontline treatment, in comparison to 18.9% of the younger patients (*p* < 0.001). However, genetic testing for germline BRCA1/2 mutations was performed in a similar manner, independent of the age of the patients (*p* = 0.108). Since BRCA1/2 testing was introduced in clinical practice in 2016, we separately analyzed patients diagnosed after 1 January 2016. Again, no statistically significant difference in BRCA1/2 testing according to age was noted (Figure S1).

### 3.3. Survival

**Figure 1:**
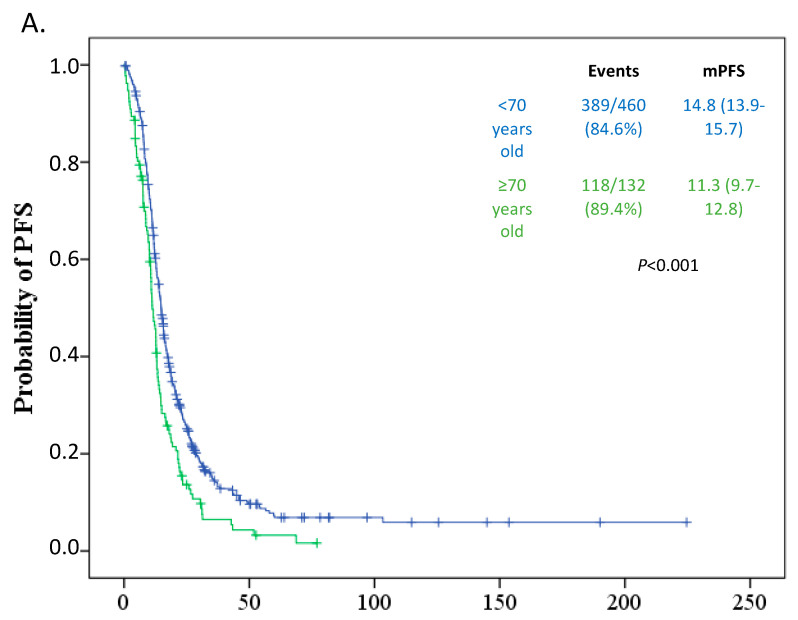

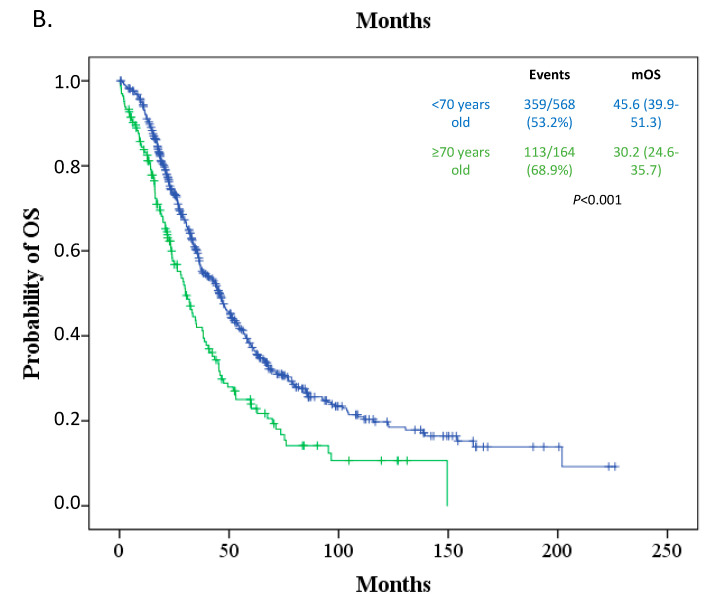
Kaplan-Meier curves showing median PFS (**A**) and OS (**B**) in patients younger (blue line) and equal to or older (green line) than 70 years of age.

Elderly patients had worse median Progression-Free Survival (PFS) (11.3 months, 95% CI 9.7–12.8) in comparison to patients younger than 70 years old (14.8 months, 95% CI 13.9–15.7) (*p* < 0.001) (Figure 1).

Furthermore, elderly patients had significantly worse Overall Survival (OS) (30.2 months, 95% CI 24.6–35.7) in comparison to their younger counterparts (45.6 months, 95% CI 39.9–51.3) (*p* = 0.011) (Figure 1). We have also tested the importance of known ovarian cancer prognostic factors regarding Progression-Free Survival (PFS) and Overall Survival (OS) in our population. In the univariate analysis, all tested parameters as age, performance status equal or greater than 2, stage IV disease at diagnosis, inability to perform primary debulking surgery, suboptimal outcome of the debulking surgery, frontline chemotherapy with carboplatin only, and omission of bevacizumab in the frontline treatment were associated with worse progression-free and overall survival (Table 2). In the multivariate analysis though, age has no independent predictive or prognostic significance. Worse performance status, inability to perform any cytoreductive surgery, suboptimal surgical outcome, and frontline chemotherapy with carboplatin only were confirmed as the independent risk factors for recurrence and death, as shown in Table 2.

## 4. Discussion

A significant percentage of high-grade serous ovarian cancer patients are diagnosed beyond 70 years of age in contrast to other ovarian cancer histologies [21]. These patients are usually underrepresented in clinical trials. Therefore, the clinicians have limited prognostic information for this group of patients so as to plan medical and surgical treatments. Currently, like the general population, elderly patients should be treated with debulking surgery and platinum-based doublet chemotherapy. However, limited data exist to guide these decisions. Furthermore, several factors related to senescence, as comorbidities, deterioration of performance status, multiple concomitant medications as well as perceptions and logistic problems related to the administration of chemotherapy in an elderly patient may hamper optimal care.

In our analysis, we have shown that elderly patients have a significantly worse prognosis than their younger counterparts. Median Overall Survival (mOS) and median Progression-Free Survival (mPFS) were far greater for patients younger than 70 years of age compared to the elderly. Our results are in accordance with previous studies which confirm the poor prognosis of elderly patients with locally advanced ovarian cancer [2,22]. However, in our multivariate analysis, age over 70 years was not an independent prognostic factor for either recurrence or death. This indicates that these disparities may be partially explained by the frailty of older patients and the differences in their treatment. Indeed, being of older age with advanced disease and concurrently with a complicated medical history that requires several concomitant medications could result in patient’s inability to receive standard surgical and systemic treatment [23].

The preferred initial treatment of these patients includes primary cytoreduction followed by systemic chemotherapy, while patients who are not candidates for primary surgery should be offered neoadjuvant chemotherapy followed by Interval Debulking Surgery [5]. Cytoreductive surgery and primary complete tumor reduction is the most important prognostic factor for advanced ovarian cancer [24,25]. Even when adjusted for other covariates, both performance of cytoreductive surgery and surgical outcome retained their significance as independent risk factors for both recurrence and death. Indeed, in our cohort of patients, those older than 70 years of age were more often unfit for surgery at all times. The surgical outcome was also associated with patients’ age, suggesting that elderly patients either are not fit for or are not offered extensive debulking surgical approaches. Several concerns exist in the published literature regarding the cost–benefit ratio of complete debulking surgery in older patients [23,26], underlying the need for a thorough evaluation of these patients prior to treatment. In addition, in accordance with the data presented here, several studies indicate the increased percentage of elderly patients subjected to interval and not primary debulking surgery [27,28]. This indicates that further research is necessary regarding optimal surgical approach among elderly patients in the era of PARP inhibitors in ovarian cancer.

Moreover, the recommended systemic chemotherapy for advanced ovarian cancer consists of a platinum-based agent, namely, carboplatin compound with a taxane; paclitaxel administered intravenously every three weeks [5], as it is shown to improve both overall and progression-free survival [29,30]. However, elder patients were more frequently unfit to receive this chemotherapy combination and were treated only with carboplatin. Recently though, the randomized trial EWOC-1 provided sufficient evidence that the paclitaxel–carboplatin combination should remain the standard of care even for older patients with ovarian cancer. This study randomized patients with advanced epithelial ovarian cancer and older than 70 years and with a Geriatric Vulnerability Score equal to or greater than 3 to receive either carboplatin monotherapy or the weekly or triweekly paclitaxel–carboplatin combination [31]. The study closed prematurely based on the Data Monitoring committee suggestion since the survival was significantly worse in the carboplatin monotherapy arm. Obviously, combinations were associated with higher toxicity and discontinuation rate due to toxicity. Other studies have also confirmed that the elderly present more frequently with severe chemotherapy-related toxicities, which often result in dose reduction and schedule delay [11,12,13] and the same applies also for maintenance bevacizumab treatment, as shown in the ROSiA trial [32].

The above-referenced differences in the surgical and medical care of elderly ovarian cancer patients could be attributed to either the true limitations of age—as previously mentioned—or the bias of the treating physicians towards elderly patients. Our site is ESGO certified for the quality of care in ovarian cancer patients, therefore the inherent bias towards treating elderly patients should be limited. Furthermore, the proportion of elderly patients not receiving optimal surgical or medical treatment does not differ significantly from published data [9]. This suggests though that the biology of the disease in elderly patients could be different and more resistant to currently applied therapies.

Furthermore, genetic counseling and testing for BRCA1/2 mutations should be offered to all newly diagnosed patients, as it was estimated that 18% of ovarian cancer cases were associated with germline mutations, mostly of the BRCA1/2 genes [6]. Patients with these mutations will benefit from maintenance treatment with polyadenosine diphosphate-ribose polymerase (PARP) inhibition [7]. In our institution, genetic testing was offered to both young and old patients alike.

Though this study has certain limitations, including its retrospective nature and differences in the surgical and medical management of patients through the observation period, it has the strong point of including a great number of patients. According to our knowledge, this is the largest study focusing on elderly ovarian cancer patients in Greece. In addition, all patients were diagnosed with advanced high-grade serous ovarian cancer and treated in a single institution ensuring uniform medical practices. These data provide useful information regarding the therapeutic areas where the management of elderly ovarian cancer patients could be improved.

## 5. Conclusions

Multiple lines of evidence suggest that high-grade serous ovarian cancer patients diagnosed over the age of 70 years have significantly worse Progression-Free Survival and Overall Survival. According to our data, suboptimal surgical management is the most important negative prognostic factor for these patients. Whether this represents a limitation of age or different biology of the disease necessitates further study. Delineating though objective means that could guide the selection of the aggressiveness of treatment in this population remains an unmet medical need.

## Figures and Tables

**Table 1:** Clinicopathological characteristics of the total population and differences in their distribution among patients older than 70 years of age.

Characteristic	Total Population	Age < 70	Age ≥ 70	
	**Median (Range)**	**Median (Range)**	**Median (Range)**	
**Age**	61.5 (24.7–89.2)	57.9 (24.7–69.9)	74.2 (70.0–89.2)	
	**N (%)**	**N (%)**	**N (%)**	***p***
**ECOG-PS**				<0.001
0–1	575 (78.2)	471 (82.6)	104 (63.0)	
≥2	111 (15.2)	56 (9.8)	55 (33.3)	
Missing	49 (6.6)	43 (7.6)	6 (3.7)	
**Stage**				0.906
III	605 (82.3)	470 (82.4)	135 (81.8)	
IV	123 (16.7)	95 (16.7)	28 (17.0)	
Missing	7 (1.0)	5 (0.9)	2 (1.2)	
**Surgery**				<0.001
PDS	578 (78.6)	461 (80.9)	117 (70.9)	
IDS	110 (15.0)	83 (14.6)	27 (16.3)	
No surgery	44 (6.0)	23 (4.0)	21 (12.7)	
Missing	3 (0.4)	3 (0.5)	0	
**Surgical outcome**				0.047
Optimal	258 (35.1)	212 (37.2)	46 (27.9)	
Suboptimal	447 (60.8)	338 (59.3)	109 (66.1)	
Missing	30 (4.1)	20 (3.5)	10 (6.0)	
**First-line chemotherapy**				<0.001
Platinum doublet	596 (81.1)	484 (84.9)	112 (67.9)	
Carboplatin	35 (4.8)	9 (1.6)	26 (15.7)	
Missing	104 (14.1)	77 (13.5)	27 (16.4)	
**Bevacizumab administration**				<0.001
Yes	121 (16.5)	108 (18.9)	13 (7.8)	
No	614 (83.5)	462 (81.1)	152 (92.2)	
**gBRCA testing**				0.108
Yes	110 (15.0)	92 (16.1)	18 (10.6)	
No	625 (85.0)	478 (83.9)	147 (89.4)	

**Table 2:** Univariate and multivariate Cox-regression analysis for PFS and OS.

	PFS						OS					
	Univariate			Multivariate			Univariate			Multivariate		
	HR	95% CI	*p*-Value *	HR	95% CI	*p*-Value	HR	95% CI	*p*-Value *	HR	95% CI	*p*-Value
**Age**			<0.001			0.075			<0.001			0.294
<70	1			1			1			1		
≥70	1.54	1.25–1.89		1.26	0.98–1.62		1.60	1.29–1.98		1.15	0.89–1.48	
**ECOG-PS**			<0.001			0.018			<0.001			<0.001
0–1	1			1			1			1		
≥2	2.00	1.59–2.53		1.42	1.06–1.90		2.64	2.09–3.35		1.80	1.37–2.38	
**Stage**			<0.001			0.182			<0.001			0.311
III	1			1			1			1		
IV	1.56	1.24–1.95		1.19	0.92–1.56		1.55	1.22–1.95		1.15	0.87–1.52	
**Surgery**			<0.001			0.007			<0.001			<0.001
PDS	1			1			1			1		
IDS	0.95	0.76–1.20		1.08	0.83–1.42		1.09	0.82–1.45		1.34	0.96–1.87	
No surgery	3.38	2.38–4.79		2.22	1.34–3.66		4.74	2.00–8.83		3.64	2.29–5.76	
**Surgical outcome**			<0.001			<0.001			<0.001			<0.001
Complete	1			1			1			1		
Optimal/Suboptimal	1.877	1.55–2.27		1.55	1.25–1.92		2.08	1.68–2.57		1.83	1.45–2.31	
**First-line chemotherapy**			0.004			0.035			<0.001			<0.001
Platinum doublet	1			1			1			1		
Carboplatin	1.73	1.19–2.50		1.62	1.03–2.52		2.46	1.68–3.59		2.19	1.43–3.38	
**Bevacizumab administration**			0.001			0.717			0.010			0.332
Yes	1			1			1			1		
No	1.45	1.16–1.82		1.19	0.92–1.56		1.47	1.09–1.98		1.21	0.83–1.76	

* Cox-regression. Missing values in Stage, Surgical outcome, ECOG-PS, First-line chemotherapy and Surgery 7, 30, 49, 80, and 3, respectively. PFS = Progression-Free Survival, OS = Overall Survival, PDS = Primary Debulking Surgery, IDS = Interval Debulking Surgery, HR = Hazard Ratio, CI = Confidence Intervals, PS = Performance Status.

## Data Availability

Data available on request due to restrictions eg privacy or ethical.

## Supplementary Materials

The following are available online at https://www.mdpi.com/1718-7729/28/2/110/s1, Figure S1: Bars indicate number of younger (blue) or elderly (red) patients that were tested or not for BRCA1/2 mutations.
