# Supplementary figures and images for: Management of the Elderly Patients with High-Grade Serous Ovarian Cancer in the REAL-WORLD Setting
Source: Curr Oncol. 2021 Mar 7;28(2):1143–52. doi: 10.3390/curroncol28020110 (PMC8025751; doi:10.3390/curroncol28020110)

Supplemental Figure 1

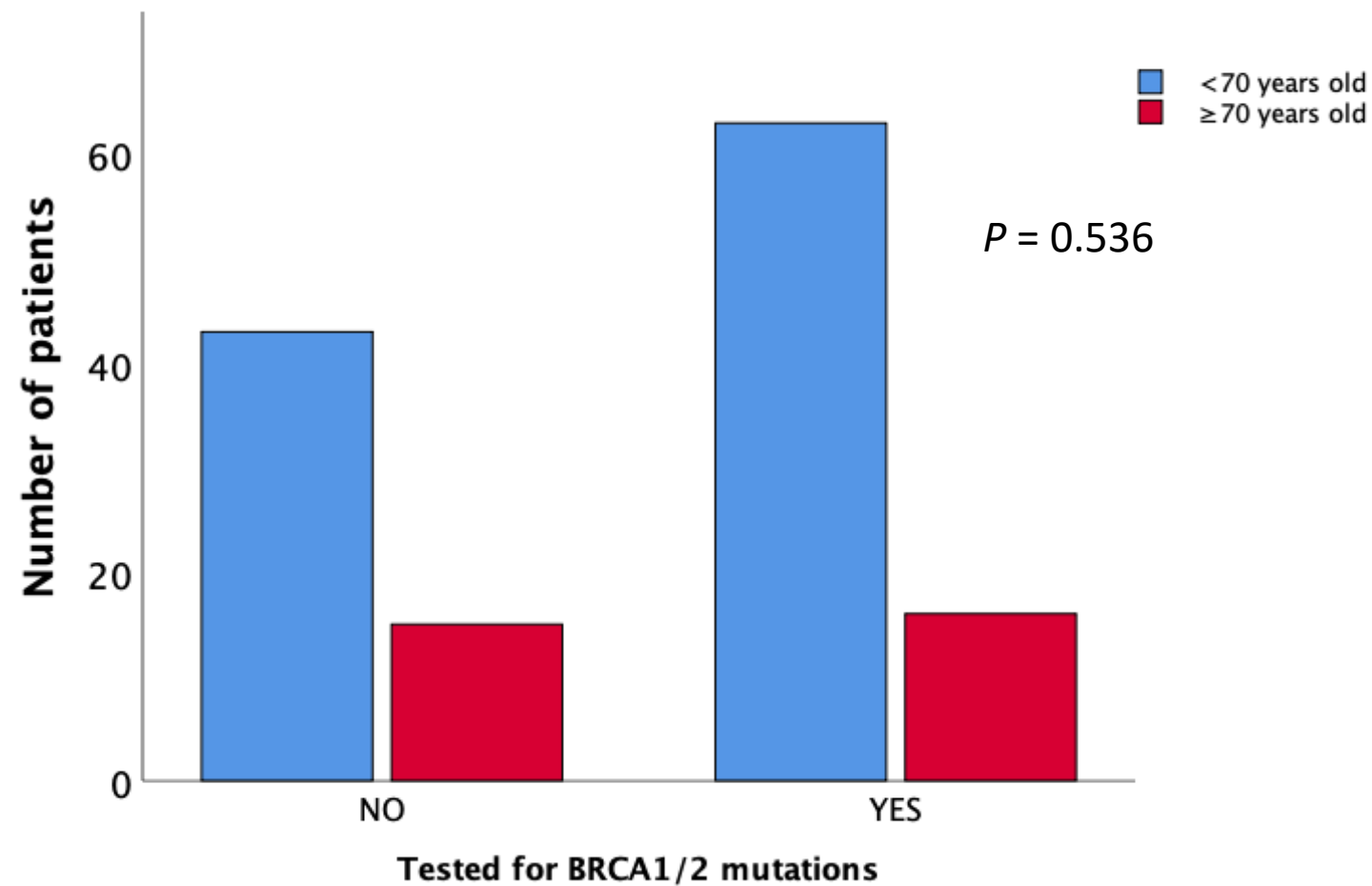

Supplement: Supplementary file 1 [file curroncol-28-00110-s001.pdf]
